# Safety of Low-calcium Dialysate and its Effects on Coronary Artery Calcification in Patients Undergoing Maintenance Hemodialysis

**DOI:** 10.1038/s41598-018-24397-w

**Published:** 2018-04-13

**Authors:** Yang Wen, Hua Gan, Zhengrong Li, Ximin Sun, Ying Xiong, Yunfeng Xia

**Affiliations:** 1 The first affiliated hospital of Chongqing Medical University, Department of Nephrology, Chongqing, 400016 China; 2 The first hospital affiliated to Army Medical University, Department of Hepatology, Chongqing, 400038 China

## Abstract

To determine the safety of low-calcium-dialysate in patients undergoing maintenance hemodialysis (MHD) and its effects on coronary artery calcification (CAC) and analyze clinical risk factors for CAC. A total of 174 MHD patients were recruited and randomly divided into two groups: high-calcium dialysate (HCD, 1.5 mmol/L Ca^2+^) and low-calcium dialysate (LCD, 1.25 mmol/L Ca^2+^). Changes in CAC score (CACS) and cardiac function were evaluated using spiral computed tomography and echocardiography, respectively. Clinical and laboratory parameters were measured. Intra-dialysis adverse reactions were recorded and compared between the two groups. CACS was significantly lower in the LCD group than in the HCD group by the end of the study. Cardiac E/A_max_ was significantly higher in the LCD group than in the HCD group by the end of the study. There was no significant difference in the frequency of any intra-dialysis adverse reactions between the two groups during the study. LCD is helpful in maintaining cardiac diastolic function and postponing CAC progression. LCD does not increase intra-dialysis adverse reactions. Age may be the most important factor impacting CAC in MHD patients.

## Introduction

The number of patients with chronic kidney disease (CKD) on maintenance hemodialysis (MHD) is increasing rapidly in China. Studies have shown that there are currently >600,000 MHD patients in China. It has been a challenge to improve the prognosis and quality of life for MHD patients. Cardiovascular diseases and events are the leading causes of death among MHD patients. According to data from the American Dialysis Registration System, >50% of MHD patients for whom the cause of death was known died from cardiovascular diseases, while the corresponding rate in China was 44.2%. Why is the incidence of cardiovascular diseases so high among MHD patients? A number of studies have shown that the cause may be secondary to cardiovascular calcification. The purpose of the current study was to determine the effects and risks of dialysates with different calcium concentrations on coronary artery calcification (CAC) in MHD patients, and to provide scientific proof for the prevention and treatment of cardiovascular calcification.

## Results

### Comparison of general baseline clinical data and laboratory indices between the groups

A total of 174 MHD patients were recruited for this study, 86 in the LCD group and 88 in the HCD group. General clinical data and basic laboratory indices at the start of the study did not differ between the groups, as indicated in Tables 1 and 2.

**Table 1:** Comparison of general clinical data before enrollment.

Variables	LCD ($\overline{x}$ ± s, n = 86)	HCD ($\overline{x}$ ± s, n = 88)	*P* value
Age (years)	52.14 ± 16.59	53.64 ± 15.39	0.448
sex ratio (M/F)	42/44	48/40	0.594
constituent of primary disease (case)*			0.807
CGN	21	19	
DN	23	26	
HRD	11	8	
Others	31	35	
dialysis age (months)	38.82 ± 24.28	42.82 ± 26.01	0.347
BMI (Kg/m^2^)	22.82 ± 2.89	22.95 ± 3.52	0.857
SBP (mmHg)	137.66 ± 11.04	137.11 ± 1.01	0.738
DBP (mmHg)	81.77 ± 7.16	82.85 ± 14.24	0.380

*Note: Constituents of primary disease, χ^2^ = 0.977, *p* = 0.807.

**Table 2:** Comparison of laboratory indices before enrollment.

Variables	LCD ($\overline{x}$ ± s, n = 86)	HCD ($\overline{x}$ ± s, n = 88)	*P* value
Ca (mmol/L)	2.39 ± 0.15	2.33 ± 0.37	0.870
P (mmol/L)	1.90 ± 0.59	1.81 ± 0.44	0.348
iPTH (pg/dl)	487.31 ± 269.56	595.96 ± 63.47	0.386
Pre dialysis Scr (umol/L)	836.70 ± 311.51	753.66 ± 255.33	0.135
Pre dialysis BUN (mg/L)	19.55 ± 10.08	18.51 ± 8.75	0.463
Alb (g/L)	40.76 ± 4.12	40.20 ± 4.14	0.214
TC (mmol/L)	3.65 ± 1.06	3.74 ± 1.37	0.810
TG (mmol/L)	1.86 ± 2.09	1.80 ± 1.15	0.238
HDL-C (mmol/L)	1.29 ± 0.43	1.18 ± 0.43	0.060
LDL-C (mmol/L)	20.83 ± 0.83	1.95 ± 1.02	0.782
Hb (g/L)	110.34 ± 13.07	107.76 ± 18.98	0.296
Serum IL-6 (pg/ml)	18.315 ± 8.23	25.88 ± 3.21	0.318
Serum TNF-a (pg/ml)	45.94 ± 27.4	50.22 ± 35.91	0.630
MDA (nmol/ml)	3.77 ± 1.72	3.90 ± 2.07	0.819
TAC (U/ml)	20.19 ± 4.84	21.38 ± 6.37	0.373
T-SOD (U/ml)	157.61 ± 4.35	158.28 ± 16.62	0.523

Note: Ca^2+^, calcium; P, phosphorus; iPTH, intact parathyroid hormone; MDA, malondialdehyde; TAC, total anti-oxidative capacity; T-SOD, total superoxide dismutase.

### Comparison of CACS before and after treatment in the two groups

Of the 174 patients, 164 completed the entire study. Of the remaining ten patients, five died during the study, three changed to peritoneal dialysis, and two went to another center. Heart spiral CT examinations revealed that the CACS before enrollment did not significantly differ between the groups. At 12 months after enrollment, CACS increased to different degrees compared to baseline in both groups; CACS was lower in the LCD than the HCD group and the difference was statistically significant (Table 3).

**Table 3:** Comparison of CACSs pre- and post-treatment in the two groups.

Items	LCD ($\overline{x}$ ± s, n = 80)	HCD ($\overline{x}$ ± s, n = 84)	*p* value
pre-treatment	289.09 ± 644.79	454.14 ± 1104.67	0.749
post-treatment	394.86 ± 809.47	816.06 ± 1548.41	0.018
ΔCACS	105.78 ± 206.37	361.92 ± 616.44	<0.001

### Comparison of pre- and post-treatment cardiac function between the groups

Ultrasound investigation revealed no significant difference between the groups with respect to left ventricular EF and E/A_max_ at the beginning of the study. By the end of the study, however, left ventricular E/A_max_ was significantly higher in the LCD group than in the HCD group (Table 4).

**Table 4:** Comparison of pre- and post-treatment cardiac function in the two groups.

Variables	LCD ($\overline{x}$ ± s, n = 80)	HCD ($\overline{x}$ ± s, n = 84)	*p* value
**pre-treatment**			
EF	0.626 ± 0.039	0.617 ± 0.083	0.375
E/Amax	0.905 ± 0.383	0.914 ± 0.111	0.845
**post-treatment**			
EF	0.627 ± 0.038	0.635 ± 0.061	0.321
E/Amax	0.833 ± 0.134	0.753 ± 0.116	<0.001

### CAC risk factor analysis

Logistic regression analysis showed that CAC occurrence was only associated with patient age, and not with dialysis duration, blood pressure, serum Ca^2+^, phosphorus (P), intact parathyroid hormone (iPTH), TNF-α, total anti-oxidative capacity (TAC), malondialdehyde (MDA), total superoxide dismutase (T-SOD), IL-6, albumin, or blood lipids (Table 5).

**Table 5:** Analysis of risk factors for CAC in MHD patients via logistic regression analysis.

variables	regression coefficient (B)	OR(Exp)	95%CI prescribed minimum	95%CI upper limit	*p* value
Age	0.089	1.094	1.047	1.142	<0.001
gender	0.182	1.199	0.32	4.492	0.787
dialysis ages	−0.011	0.989	0.968	1.009	0.283
BMI	0.157	1.170	1.004	1.364	0.055
SBP (mmHg)	0.014	1.014	0.965	1.064	0.587
DBP (mmHg)	−0.005	0.995	0.946	1.047	0.844
Pre-dialysis BUN (mg/L)	0.016	1.017	0.958	1.079	0.585
Pre-dialysis Scr (umol/L)	−0.001	0.999	0.997	1.001	0.529
Ca	1.283	3.609	0.466	27.976	0.219
P	0.582	1.789	0.719	4.455	0.211
iPTH	0.000	1.000	0.999	1.001	0.676
TNF-α	−0.011	0.989	0.976	1.001	0.080
MDA	0.195	1.216	0.936	1.579	0.143
TAC	0.067	1.069	0.981	1.165	0.129
T-SOD	−0.010	0.990	0.956	1.026	0.598
IL-6	0.002	1.002	0.994	1.010	0.592
ALB (g/L)	0.053	1.055	0.947	1.174	0.330
TC (mmol/L)	0.337	1.401	0.844	2.325	0.192
TG (mmol/L)	−0.074	0.928	0.678	1.271	0.643
HDL-C (mmol/L)	−0.070	0.932	0.283	3.066	0.908
LDL-C (mmol/L)	−0.187	0.829	0.472	1.457	0.516
Hb (g/L)	−0.001	0.999	0.971	1.028	0.946

Note: TAC, total anti-oxidative capacity, MDA, malondialdehyde, T-SOD, total superoxide dismutase.

### Comparison of intra-dialysis adverse reactions between the groups

There was no significant difference in intra-dialysis hypotension, hypertension, muscle cramps, or arrhythmias between the groups. By the end of the study, however, serum Ca^2+^ was significantly lower and serum iPTH was significantly higher in the LCD group than in the HCD group (Table 6).

**Table 6:** Comparison of the incidence of adverse reactions between the groups.

Items	LCD (n = 80)	HCD (n = 84)	*p* value
Intradialytic hypotension (person-time)	704	813	0.066^a^
Intradialytic hypertension (person-time)	6083	6530	0.137^b^
Muscle cramp (person-time)	210	198	0.259^c^
Arrhythmia (person-time)	1873	1948	0.668^d^
Total person-time	12405	13068	/

Note: ^a^chi-square test, *χ*^2^ = 3.390, *p* = 0.066; ^b^chi-square test, *χ*^*2*^ = 2.215, *p* = 0.137; ^c^ chi-square test, *χ*^*2*^ = 1.275, *p* = 0.259; ^d^chi-square test, *χ*^*2*^ = 0,184, *p* = 0.668.

## Discussion

Cardiovascular events are the primary threat to the safety of MHD patients^1,2^, whose risk of cardiovascular death is 10–20 times that of the general population^3,4^. Vascular calcification is an independent risk factor for cardiovascular events in patients with CKD^5^. The incidence of vascular calcification increases with CKD progression^6^. Elevated serum P and long-term Ca^2+^ overload are the main causes of vascular calcifications in patients^7–10^. For MHD patients, the impact of the Ca^2+^ concentration in dialysate on Ca^2+^ balance cannot be ignored. Long-term use of HCD with a Ca^2+^ ion concentration of 1.5 or 1.75 mmol/L promotes a positive Ca^2+^ balance; however, long-term use of HCD increases the risk of vascular calcification in patients^8,10^. Studies have suggested that long-term use of dialysate containing 1.25 mmol/L Ca^2+^ can effectively reduce the risk of cardiovascular calcification in MHD patients^11–13^, but it may also increase the risk of hypotension, muscle twitching, arrhythmias, and even sudden cardiac death in patients during dialysis^14–16^.

At present, HCD containing 1.5 mmol/L Ca^2+^ is used most frequently at most blood purification centers in China because many patients and physicians at primary hospitals are still reluctant to use dialysate with 1.25 mmol/L Ca^2+^ because it may reduce serum Ca^2+^ or increase the risk of adverse reactions, such as hypotension, muscle convulsions, and arrhythmias during dialysis. In the current study, we investigated the effects of two types of dialysate containing 1.5 or 1.25 mmol/L Ca^2+^ on CAC and possible adverse reactions in MHD patients during conventional hemodialysis (3 times per week for 3.5–4 hours each time). The results show that CACSs in patients on long-term LCD were significantly lower than in the HCD group, even though CACSs increased to a different extent in both groups after 12 months of treatment. Cardiac ultrasound also showed significantly higher cardiac E/A_max_ values in the LCD than in the HCD group at the end of the study, suggesting that long-term use of LCD containing 1.25 mmol/L Ca^2+^ is more conducive to delaying CAC progression in MHD patients and is more beneficial for maintaining diastolic function than long-term HCD use. It is noteworthy that no differences were observed in the effect of the two dialysates on the incidence of hypotension, hypertension, muscle spasms, and arrhythmias during dialysis in the current study; however, long-term use of dialysates containing 1.25 mmol/L Ca^2+^ reduced serum Ca^2+^ levels in patients and stimulated an increase in serum iPTH levels.

We also analyzed common risk factors for CAC in MHD patients. The results show that CAC incidence in MHD patients was negatively correlated with age, and there was no correlation with hemodialysis duration, time-averaged blood pressure, or levels of serum Ca^2+^, serum P, serum iPTH, albumin, blood lipids, serum inflammatory indicators (TNF-α and IL-6), and serum oxidative markers (serum TAC, MDA, and T-SOD).

A number of previous studies have suggested that the occurrence of vascular calcification in MHD patients may be affected by multiple factors, such as blood pressure, serum Ca^2+^, serum P, serum iPTH, dialysis duration, malnutrition, chronic inflammatory conditions, and oxidative load^17–19^, in addition to being correlated with age. Nevertheless, results from various clinical studies have often shown differences. The reasons for these differences may be attributed mainly to the following: (i) the course of vascular calcification in MHD patients is relatively slow, and the speed at which the condition develops is closely related to the risk factors and control measures specific to an individual patient, so the observation period in a study must be sufficiently long to reveal problems; (ii) the effects of various clinical treatment measures on the parameters observed in patients, such as blood pressure, serum Ca^2+^, serum P, and serum iPTH, cannot be ignored; (iii) results from a limited observation period may not accurately reflect long-term levels of the parameters in patients; and (iii) current clinical studies on the risk factors for vascular calcification in MHD patients are mostly observational studies, with only a few randomized controlled studies with small sample sizes.

Low-calcium dialysis reduces the calcium burden in MHD patients. Low-calcium dialysis is not only conducive to the prevention and treatment of vascular calcification but also stimulates parathyroid hormone secretion and reduces the risk of low-transport bone disease^20,21^, which is the most common renal osteopathy, with an incidence of 40–70% among MHD patients^22,23^. There has long been concern about low-calcium dialysis, even though 1.25 mmol/L calcium is within the normal physiological range. There are still concerns regarding the higher risk of intra-dialysis hypotension and muscle spasms caused by low-calcium dialysis. In addition, serum Ca^2+^ concentrations have an important impact on myocardial cell excitement and contraction^24^, and long-term hypercalcemia may reduce left ventricular diastolic function in patients^25^. Studies have reported that the use of dialysate containing 1.25 mmol/L calcium induced a rapid reduction in serum Ca^2+^ concentrations in patients and affected the ventricular repolarization process, resulting in a prolonged QT interval, dispersion, arrhythmias, and even sudden cardiac death in severe cases^15,26–28^. In the current study, we closely monitored patients for intra-dialysis hypotension, hypertension, muscle spasms, and arrhythmias in the two groups, and found no differences in the effect of the two types of dialysate on the occurrence of various intra-dialysis adverse reactions.

## Methods

### Patient selection

MHD patients were recruited from the Blood Purification Center of our hospital from those who met the following inclusion criteria: (i) serum calcium (Ca^2+^) ≥2.10 mmol/L and intact parathyroid hormone (iPTH) <600 pg/mL; (ii) MHD duration ≥12 months; and (iii) stable health, compliant with treatment, and active following treatment. The exclusion criteria were as follows: (i) concomitant chronic respiratory failure or long-term cardiac functional grade III–IV; (ii) concomitant cirrhosis, tuberculosis, tumors, AIDS, or disease affecting prognosis; and (iii) unable or declined to participate in the study. The study protocol was approved by the Ethics Committee of the first affiliated hospital of Chongqing Medical University and informed consent was obtained from all participants. CLINICAL TRIAL registration number: NCT02498457. We confirm that all experiments of the study were performed in accordance with relevant guidelines and regulations.

### General clinical data and measurement of laboratory indices

General clinical data were collected and laboratory indices were measured during the study for all patients, including age, gender, time on hemodialysis, blood pressure, primary disease, body mass index, Ca^2+^, phosphorus (P), iPTH, hemoglobin (Hb), and blood lipids. The concentrations of serum IL-6 and TNF-α were measured using an enzyme-linked immunosorbent assay (ELISA; Boster, Wuhan, Hubei, China). Serum total anti-oxidative capacity (TAC) was measured by colorimetry. Total superoxide dismutase (T-SOD) was measured via the xanthine oxidase method. Serum malondialdehyde (MDA) was measured using the thiobarbituric acid method with test kits from Nanjing Jiancheng Bioengineering (Nanjing, China) according to the manufacturer’s instructions.

### Evaluation and follow-up of CAC and cardiac function

Using a spiral CT scan (Siemens SOMATOM Definition, Munich, Germany), the coronary arteries of all patients before and after the study, including the left main (LM), left anterior descending branch (LAD), left circumflex (LCX), and right coronary artery (RCA), were assessed. According to the calcification volume, the coronary artery calcification score (CACS) was evaluated using CaScoring software. At least three cardiac cycles per time were determined using echocardiography to evaluate changes in cardiac function and to collect primary indicators, including left ventricular ejection fraction (EF) and the ratio of early diastolic E to late diastolic A (E/A_max_). The results were averaged.

### Grouping and intervention

All patients were treated with high-calcium dialysate (HCD) before the study. After recruitment, the patients were randomly assigned to low-calcium dialysate (LCD; 1.25 mmol/L Ca^2+^) or HCD (1.5 mmol/L Ca^2+^). Both groups underwent hemodialysis for 3.5–4 hours three times per week for 12 months.

### Monitoring and recording adverse reactions related to dialysis

Adverse reactions related to dialysis during the study were monitored and recorded; they included hypertension, hypotension, muscle cramps, and arrhythmias. The diagnostic criteria for intra-dialysis hypertension were as follows: an increase in mean arterial pressure of >15 mmHg compared to baseline with or without dizziness, sweating, palpitations, or muscle spasms. The diagnostic criteria for intra-dialysis hypotension were as follows: a decrease in systolic blood pressure of >20 mmHg or a decrease in mean arterial pressure of >10 mmHg during or immediately after dialysis, with clinical symptoms and a need for intervention.

### Statistical analysis

Results for continuous data are presented as the mean ± standard deviation. Mean values were compared between the groups using an independent-sample t-test for normally distributed data and a non-parametric test for non-normally distributed data. Rate comparisons were performed using a *χ*^2^ test. CAC risk factors were analyzed using binary logistic regression analysis, and the binary classification of dependent variables were CACS >0 and CACS = 0, and the method was “enter”. Adverse reactions were compared between the groups using a *χ*^2^ test. Differences between the groups were significant at *p* < 0.05.

### Data availability

The datasets generated and analysed during the current study are available from the corresponding author on reasonable request^29,30^.

## Electronic supplementary material

Supplementary Information
