# Supplementary material for: Safety of Low-calcium Dialysate and its Effects on Coronary Artery Calcification in Patients Undergoing Maintenance Hemodialysis
Source: Sci Rep. 2018 Apr 13;8:5941. doi: 10.1038/s41598-018-24397-w (PMC5899126; doi:10.1038/s41598-018-24397-w)
Supplement: Supplementary file 1 — Supplementary Information [file 41598_2018_24397_MOESM1_ESM.docx]

**Safety of Low-calcium Dialysate and its Effects on Coronary Artery Calcification in Patients Undergoing Maintenance Hemodialysis**

**Yang Wen^1^, Hua Gan^1^, Zhengrong Li^1^, Ximin Sun^2^, Ying Xiong^1^, and Yunfeng Xia**^1^**^*^**

^1^ The first affiliated hospital of Chongqing Medical University, Department of Nephrology, Chongqing, 400016, China.

^2^ The first affiliated hospital of the Army Medical University, Department of Hepatology, Chongqing, 400038, China.

^*^corresponding author, Correspondence and requests for materials should be addressed to Yunfeng Xia. ([yunfengxia0920@126.com](mailto:yunfengxia0920@126.com))

| Variables | LCD（$\overline{x}$±s，n=80） | HCD（$\overline{x}$±s，n=84） | *P* value |
| --- | --- | --- | --- |
| mean SBP(mmHg) | 123.51 ± 14.66 | 126 ± 10.46 | 0.155 |
| menn DBP(mmHg) | 76.99 ± 8.52 | 77.04 ± 5.38 | 0.962 |
| Ca (mmol/L) | 1.93 ± 0.15 | 2.25 ± 0.14 | <0.001 |
| P (mmol/L) | 1.86 ± 0.45 | 1.85 ± 0.47 | 0.727 |
| iPTH (pg/dl) | 470.61 ± 181.89 | 420.89 ± 378.83 | 0.001 |
| Hb (g/L) | 111.72 ± 12.7 | 109.74 ± 11.66 | 0.110 |
| Alb (g/L) | 40.07 ± 4 | 39.87 ± 3.85 | 0.738 |
| TC (mmol/L) | 3.63 ± 1.40 | 3.36 ± 1.22 | 0.069 |
| TG (mmol/L) | 1.81 ± 0.15 | 1.75 ± 0.26 | 0.074 |
| HDL-C (mmol/L) | 1.22 ± 0.45 | 1.25 ± 0.42 | 0.554 |
| LDL-C (mmol/L) | 1.98 ± 0.1 | 1.97 ± 0.16 | 0.895 |
| Elemental Ca(g/d) | 0.72 ± 0.43 | 0.71 ± 0.39 | 0.868 |
| VitaminD (μg/wk) | 0.61 ± 0.1 | 0.38 ± 0.23 | <0.001 |

Supplementary Table S1. Time-averaged data during the 12-month study. Note: SBP, systolic blood pressure; DBP, diastolic blood pressure; Alb, albumin; TC, total cholesterol; TG, total triglycerides.
